# Extracerebral microvascular dysfunction is related to brain MRI markers of cerebral small vessel disease: The Maastricht Study

**DOI:** 10.1007/s11357-021-00493-0

**Published:** 2021-11-23

**Authors:** Maud van Dinther, Miranda T. Schram, Jacobus F. A. Jansen, Walter H. Backes, Alfons J. H. M. Houben, Tos T. J. M. Berendschot, Casper G. Schalkwijk, Coen D. A. Stehouwer, Robert J. van Oostenbrugge, Julie Staals

**Affiliations:** 1 Department of Neurology, Maastricht University Medical Center, Maastricht, The Netherlands; 2 CARIM - School for Cardiovascular Diseases, Maastricht University, Maastricht, The Netherlands; 3 Department of Internal Medicine, Maastricht University Medical Center, Maastricht, The Netherlands; 4 MHeNs - School for Mental Health and Neuroscience, Maastricht University, Maastricht, The Netherlands; 5 Department of Radiology and Nuclear Medicine, Maastricht University Medical Center, Maastricht, The Netherlands; 6 Department of Ophthalmology, Maastricht University Medical Center, Maastricht, The Netherlands; 7 NUTRIM – School of Nutrition and Translational Research in Metabolism, Maastricht University, Maastricht, The Netherlands

**Keywords:** Cerebral small vessel disease, Microcirculation, Microvascular dysfunction, White matter hyperintensities

## Abstract

**Background:**

Cerebral small vessel disease (cSVD) is a late consequence of cerebral microvascular dysfunction (MVD). MVD is hard to measure in the brain due to its limited accessibility. Extracerebral MVD (eMVD) measures can give insights in the etiology of cerebral MVD, as MVD may be a systemic process. We aim to investigate whether a compound score consisting of several eMVD measures is associated with structural cSVD MRI markers.

**Methods:**

Cross-sectional data of the population-based Maastricht Study was used (*n* = 1872, mean age 59 ± 8 years, 49% women). Measures of eMVD included flicker light-induced retinal arteriolar and venular dilation response (retina), albuminuria and glomerular filtration rate (kidney), heat-induced skin hyperemia (skin), and plasma biomarkers of endothelial dysfunction (sICAM-1, sVCAM-1, sE-selectin, and von Willebrand factor). These measures were standardized into *z* scores and summarized into a compound score. Linear and logistic regression analyses were used to investigate the associations between the compound score and white matter hyperintensity (WMH) volume, and the presence of lacunes and microbleeds, as measured by brain MRI.

**Results:**

The eMVD compound score was associated with WMH volume independent of age, sex, and cardiovascular risk factors (St *β* 0.057 [95% CI 0.010–0.081], *p* value 0.01), but not with the presence of lacunes (OR 1.011 [95% CI 0.803–1.273], *p* value 0.92) or microbleeds (OR 1.055 [95% CI 0.896–1.242], *p* value 0.52).

**Conclusion:**

A compound score of eMVD is associated with WMH volume. Further research is needed to expand the knowledge about the role of systemic MVD in the pathophysiology of cSVD.

**Supplementary Information:**

The online version contains supplementary material available at 10.1007/s11357-021-00493-0.

## Introduction

Cerebral small vessel disease (cSVD) is an umbrella term that covers all pathologies of cerebral small vessels. The most prevalent form is age- and cardiovascular risk factor associated cSVD [1]. It may cause hemorrhagic stroke, ischemic lacunar stroke, vascular cognitive impairment, and contributes majorly to dementia. Typical cSVD lesions as detected with brain magnetic resonance imaging (MRI) are white matter hyperintensities (WMH), lacunes, perivascular spaces, and microbleeds [2]. These MRI features support the diagnosis of cSVD [3, 4]. These macrostructural MRI visible lesions are assumed to be the ultimate consequence of long-standing microvascular dysfunction [5]. Microvascular dysfunction itself is hard to measure in the brain, since the advanced functional MRI techniques that can provide cerebral microvascular function measures [6] are expensive and are not widely available for large scale in population-based studies.

It is assumed that microvascular dysfunction in the brain is part of systemic microvascular dysfunction. As such, associations have already been demonstrated between individual measures of dysfunction in different extracerebral microvascular beds, such as the retina, kidney, and plasma biomarkers of endothelial dysfunction, and macrostructural brain MRI markers [7–10]. However, these individual measures have not been studied in parallel in the same population, nor have they been studied in combination. A summarizing measure of multiple vascular beds is believed to better reflect systemic microvascular dysfunction than single organ measures. Recently, we have shown a ‘microvascular dysfunction composite score’ to be associated with lower cognitive function [11]. This composite score included both extracerebral measures and cerebral MRI features. However, whether overall extracerebral microvascular dysfunction (eMVD) is actually related to structural cerebral markers of SVD has not been studied yet.

Therefore, we aim to investigate whether a compound score consisting of several measures of microvascular dysfunction in extracerebral vascular beds (the retina, kidney, skin, and plasma biomarkers) is associated with structural MRI markers of cSVD in a large population-based cohort.

## Methods

### Study population

We used data from The Maastricht Study, an observational prospective population-based cohort study. The rationale and methodology have been described previously [12]. In brief, the study focuses on the etiology, pathophysiology, complications, and comorbidities of type 2 diabetes mellitus (T2DM) and is characterized by an extensive phenotyping approach. Eligible for participation were all individuals aged between 40 and 75 years and living in the southern part of the Netherlands. Participants were recruited through mass media campaigns and from the municipal registries and the regional Diabetes Patient Registry via mailings. Recruitment was stratified according to known T2DM status, with an oversampling of individuals with T2DM for reasons of efficiency. The present report includes cross-sectional data from the first 3451 participants, who completed the baseline survey between November 2010 and September 2013. All examinations, except brain MRI, of each participant were performed within a time window of three months. Brain MRI was implemented in a second visit from December 2013 onward until February 2017. The study has been approved by the institutional medical ethical committee (NL31329.068.10) and the Ministry of Health, Welfare and Sports of the Netherlands (131,088–105,234-PG). All participants gave written informed consent. All procedures followed were in accordance with institutional guidelines.

### Extracerebral microvascular function measures

All participants were asked to refrain from smoking and drinking caffeine-containing beverages three hours before the measurements. A light meal was allowed if taken at least 90 min prior to the start of the measurements. The methodology of the extracerebral microvascular function measures has extensively been described previously [13].

#### Flicker light-induced retinal arteriolar and venular dilation response

We measured retinal arteriolar and venular dilatation response to flicker light exposure by the Dynamic Vessel Analyzer (DVA) (Imedos, Jena, Germany), as previously described [13, 14]. Briefly, vessel diameter was automatically and continuously measured for 150 s. A baseline recording of 50 s was followed by 40 s flicker light exposure, followed by a 60 s recovery period. Baseline retinal arteriolar and venular diameter and flicker light-induced retinal arteriolar and venular dilation were automatically calculated with the integrated DVA software. Baseline diameter (expressed in measurement units (MU)) was calculated as the average diameter size of the 20–50 s recording. Percentage dilation over baseline was based on the average dilation achieved at time points 10 and 40 s during the flicker stimulation period.

#### Heat-induced skin hyperemia

We measured heat-induced skin hyperemia by laser-Doppler flowmetry (Perimed, Järfälla, Sweden), as previously described [13, 14]. Briefly, the laser Doppler output was recorded for 25 min with a sample rate of 32 Hz, which gives semi-quantitative assessment of skin blood flow expressed in arbitrary perfusion units (PU). Skin blood flow at the wrist was first recorded unheated for 2 min to serve as a baseline. After 2 min, the temperature of the laser-Doppler probe was rapidly and locally increased to 44 °C, and was kept constant until the end of the registration. The heat-induced skin hyperemic response was expressed as the percentage increase in average PU during the 23 min heating phase over the 2 min average baseline PU.

#### Kidney microvascular function

Glomerular filtration rate (GFR) was estimated with the CKD-EPI equation based on serum creatinine. Serum creatinine was measured with a Jaffé method traceable to the isotope-dilution mass spectrometry (due to a change of supplier, 2 instruments were used in the study, the Beckman Synchron LX20 and the Roche Cobas 6000).

We assessed urinary albumin excretion (UAE) in two 24-h urine samples, as previously described [13, 15]. Briefly, urinary albumin concentration was measured with a standard immunoturbidimetric assay by an automatic analyzer (due to a change of supplier, by the Beckman Synchron LX20 and the Roche Cobas 6000) and multiplied by collection volume to obtain 24-h UAE. A urinary albumin concentration below the detection limit of the assay was set at 1.5 mg/L (2 mg/L for the Beckman Synchron LX20 and 3 mg/L for the Roche Cobas 6000) before multiplying by collection volume. Only urine collections with a collection time between 20 and 28 h were considered valid. If needed, UAE was extrapolated to 24-h excretion. For this study, UAE was preferably based on the average of two 24-h urine collections.

#### Plasma biomarkers of microvascular dysfunction

We measured four plasma biomarkers of microvascular disease: soluble intercellular adhesion molecule-1 (sICAM-1), soluble vascular adhesion molecule-1 (sVCAM-1), soluble E-selectin (sE-selectin), and von Willebrand factor (vWF). sICAM-1, s-VCAM-1, and sE-selectin were measured in EDTA plasma samples with commercially available 4-plex sandwich immunoassay kits with different standards and antibodies (Meso Scale Discovery, Rockville, Maryland). For this technique in this study, the intra- and inter-assay coefficients of variation were 10.3 and 8.4% for sICAM-1, 5.0 and 4.7% for sVCAM-1, and 2.9 and 7.4% for sE-selectin, respectively. vWF was quantified in citrate plasma using ELISA (Dako, Glostrup, Denmark). The intra- and inter-assay coefficients of variation were 3.0 and 4.3%, respectively.

### Structural MRI markers of cSVD

Brain MRI was performed on a 3 T MRI scanner (Siemens Magnetom Prisma-fit Singo MR D13D Erlangen, Germany) by use of a 64-element head coil for parallel imaging. The MRI protocol consisted of a 3D T1-weighted sequence, a fluid-attenuated inversion recovery (FLAIR) sequence, a combined proton density and T2-weighted turbo spin-echo sequence, and a susceptibility-weighted imaging (SWI) sequence [16]. The protocols for MRI acquisition and analysis were in line with STRIVE (STandards for ReportIng Vascular changes on nEuroimaging) V1 imaging standards [2]. The details of the MRI sequences are provided in item S1 in the data supplement. T2-weigthed FLAIR and T1-weighted images were analyzed by use of an ISO-13485:2012 certified, automated method (which included visual inspection) [17, 18] and were used to measure WMH volume. Lacunar infarcts were defined as focal lesions of ≥ 3 mm and ≤ 15 mm in size with a signal intensity similar to that of the cerebrospinal fluid (CSF) on all sequences and a hyperintense rim on T2 and FLAIR images. Cerebral microbleeds were rated on 3D T2*-SWI by use of the Microbleed Anatomical Rating Scale [19] and were defined as focal lesions of ≥ 2 mm and ≤ 10 in size with a hypointense signal. The number and location of lacunar infarcts and cerebral microbleeds were rated manually by three neuroradiologists. The interclass correlation coefficient (95% CI) for the three raters based on 50 randomly selected scans was 0.84 (0.74–0.91) and 0.83 (0.72–0.90) for the presence of lacunar infarcts and microbleeds, respectively.

### General characteristics and covariates

We determined diabetes mellitus status by use of an oral glucose tolerance test, defined according to the World Health Organization 2006 criteria into normal glucose metabolism, prediabetes, or type 2 diabetes mellitus [12]. Fasting plasma glucose, HbA1c levels, smoking status (never, former, current), body mass index (BMI), waist circumference, office blood pressure, 24-h ambulatory blood pressure (Watch BP03; Microlife AG, Widnau, Switzerland), plasma lipid levels, prior cardiovascular disease, medication use, alcohol use (non-consumers, low-consumers, high-consumers), healthy diet (Dutch Healthy Diet (DHD) index), and self-reported physical activity (Community Health Activities Model Program for Seniors physical activity (CHAMPS) questionnaire) were determined as described previously [12, 14, 20].

### Statistical analysis

Characteristics of the study population were presented as mean ± standard deviation (SD), as median (interquartile range), or as percentages. WMH volume was log10-transformed after adding 0.0005 ml to normalize its skewed distribution. We converted UAE into a categorical variable (< 15, 15– < 30 and ≥ 30 mg/24 h) [21], since UAE and WHM volume were not linearly associated. We inversed retinal arteriolar and venular dilation response, GFR, and heat-induced skin hyperemia, so higher values indicated more severe microvascular dysfunction. For calculation of the compound score, we standardized all individual eMVD measures (retinal arteriolar and venular dilation response, UAE, GFR, heat-induced skin hyperemia, sICAM-1, s-VCAM-1, sE-selectin, and vWF) into *z* scores. Subsequently, we averaged these *z* scores into one score for each organ (retina, kidney, skin, and plasma biomarkers) and standardized these average scores into *z* scores (e.g. retina = *z* score of (*z* score retinal arteriolar dilation response + *z* score retinal venular dilation response)/2). These four single organ *z* scores were averaged into the compound score, which was finally standardized into a *z* score as well (i.e. eMVD compound score = *z* score of ((*z* score retina + *z* score kidney + *z* score skin + *z* score plasma biomarkers) /4)). We only included participants who had eMVD measures of at least three out of four organs available. Presence of only arterial or venous dilation response, only GFR or albuminuria, and at least three out of four plasma biomarkers were considered sufficient for representation of the corresponding organ in the compound score.

We used linear (WMH volume) and logistic (presence of lacunes and microbleeds) regression analysis to determine the association between the compound score and cSVD MRI markers. Model 1 was adjusted for age, sex, and the time between the eMVD measurements and MRI measurement. Model 2 was additionally adjusted for smoking status, BMI, diabetes mellitus status, office systolic blood pressure, use of antihypertensives, total/HDL cholesterol ratio, and lipid-modifying medication. For analysis of the association with WMH volume, additional adjustment for intracranial volume was included (both in model 1 and model 2).

We also analyzed each individual eMVD organ measure (averaged *z* score for each individual organ) as the determinant to test whether the association between the compound score and each cSVD marker was primarily determined by microvascular dysfunction of individual organ beds.

Furthermore, several additional analyses were performed using model 2 to study result robustness. (i) We repeated the regression analyses and subsequently substituted (a) diabetes status for HbA1c + glucose-lowering medication and fasting plasma glucose + glucose-lowering medication, (b) office blood pressure for 24-h ambulatory blood pressure (*n* = 227 missings), and (c) BMI for waist circumference. (ii) We additionally corrected for alcohol use, diet, and physical activity. (iii) For calculation of the retinal component of the eMVD compound score, we replaced flicker light-induced retinal arteriolar and venular dilation response with retinal vessel diameters, i.e. central retinal arteriolar equivalent (CRAE) and central retinal venular equivalent (CRVE), and repeated linear and logistic regression analysis (the details of the methodology of acquiring these retinal vessel diameters are provided in item S2 in the data supplement). (iv) We repeated logistic regression analysis for microbleeds with any deep, and then any lobar cerebral microbleeds as the outcome variable.

All analyses were performed with the SPSS software (v26.0; IBM, Chicago). A *p* value of < 0.05 was considered statistically significant.

## Results

### General characteristics of the study population

Brain MRI was available in 2313 of 3451 participants. Eleven MRI scans were excluded owing to pathology (*n* = 2), metal artifacts (*n* = 1), or insufficient scan quality (*n* = 8). Participants with eMVD measures of less than three organ beds available (*n* = 430) were excluded from the analysis. The remaining 1872 participants all had data available on all covariates used in model 1; all additional covariates used in model 2 were available in 1846 participants (Supplementary Figure S1).

Table 1 shows the general characteristics of the study population. The mean age was 59 ± 8 years, and 49% were women. Individuals who underwent MRI and had at least 3 eMVD measures available were younger and were less likely to have diabetes mellitus and other cardiovascular risk factors, than the 1579 non-included participants (Supplementary Table S1).

**Table 1:** Characteristics of the study population

Characteristic	Study population (*N* = 1872)
General characteristics	
Age [years]	59 ± 8
Sex [% women]	48.8 (914)
BMI [kg/m^2^]	26.5 ± 4.2
Diabetes status [% none/prediabetes/type 2 diabetes/other types diabetes]	59.9/15.7/23.1/1.4 (1121/293/432/26)
Hypertension %	52.5 (982)
Office systolic blood pressure [mmHg]	134 ± 17
Blood pressure lowering medication %	35.5 (664)
Smoking, never/former/current %	37.5/50.9/11.6 (693/940/214)
Total/HDL cholesterol ratio	3.6 ± 1.2
Lipid modifying medication %	31.5 (590)
Alcohol use, none/low/high %	17.1/56.3/26.6 (316/1042/492)
Physical activity [h/week]	14 ± 8
DHD index	84 ± 14
History of cardiovascular disease %	11.8 (217)
History of stroke %	1.9 (35)
Extracerebral microvascular dysfunction measures	
Flicker light-induced arteriolar dilation response [%]	3.2 ± 2.8
Flicker light-induced venular dilation response [%]	3.9 ± 2.2
Albuminuria [< 15/15– < 30/ ≥ 30 mg/24 h] %	83.7/9.2/7.1 (1556/172/132)
eGFR [ml/min/1.73m^2^]	85 ± 13
Heat-induced skin hyperemia [%]	1154 ± 785
sICAM-1 [µg/l]	350 ± 91
sVCAM-1 [µg/l]	427 ± 98
E-selectin [µg/l]	113 ± 62
vWF [%]	130 ± 48
cSVD imaging characteristics	
Total intracranial volume [ml]	1389 ± 135
WMH volume [ml]	0.23 (0.07–0.73)
Relative WMH volume ≥ 3%	0.4 (7)
Cerebral microbleeds %	12 (219)
Lacunes %	5.2 (96)

Data are presented as means ± SD, median (interquartile range), or percentages of participants (*n*)

*BMI*, body mass index; *HDL*, high density lipidprotein; *DHD index*, Dutch Healthy Diet index; *eGFR*, estimated glomerular filtration rate; *sICAM-1*, soluble intercellular adhesion molecule-1; *sVCAM*-*1*, soluble vascular adhesion molecule-1; *sE-selectin*, soluble E-selectin; *vWF*, von Willebrand factor; *SD*, standard deviation

### Extracerebral microvascular dysfunction and WMH volume

The eMVD compound score was significantly associated with larger volumes of WMHs (Table 2). The association remained significant after adjustment for sex, age, time between the eMVD measurements and MRI measurement, and intracranial volume (model 1: St *β* 0.099 [95% CI 0.046–0.113]; *p* < 0.01), and after additional adjustment for cardiovascular risk factors (model 2: St *β* 0.057 [95% CI 0.010–0.081]; *p* = 0.01). Full results of model 1 and model 2, including all regression coefficients for all confounders, are provided in supplementary tables S2 and S3 respectively. None of the individual organ bed eMVD scores was statistically significantly associated with WMH volume after adjustment for age, sex, and major cSVD risk factors (Table 3).

**Table 2:** Associations of the extracerebral microvascular dysfunction compound score and macrostructural MRI markers of cSVD

eMVD compound score	WMH volume		Lacunes		Microbleeds	
	St *β* (95%CI)	*p* value	OR (95%CI)	*p* value	OR (95%CI)	*p* value
Unadjusted	0.241 (0.197–0.285)	< 0.01	1.361 (1.124–1.647)	< 0.01	1.247 (1.086–1.431)	< 0.01
Model 1	0.099 (0.046–0.113)	< 0.01	1.096 (0.886–1.355)	0.40	1.074 (0.924–1.248)	0.35
Model 2	0.057 (0.010–0.081)	0.01	1.011 (0.803–1.273)	0.92	1.055 (0.896–1.242)	0.52

Associations of the microvascular dysfunction compound score and structural brain abnormalities in the study population. Point estimates (standardized *β*) and 95% CIs indicate the mean difference in WMH volume (in log10-transformed ml) per SD higher microvascular compound score. Odds ratios with 95% CI represent the risk of the presence of lacunes or cerebral microbleeds

*WMH*, white matter hyperintensity volume; *eMVD*, extracerebral microvascular dysfunction; *CI*, confidence interval; *OR*, odds ratio, *SD* standard deviation

Model 1: adjustment for sex, age, and time between the extracerebral microvascular function measures and MRI measurement

Model 2: model 1 additionally adjusted for BMI, diabetes status, office systolic blood pressure, use of antihypertensives, total/HDL cholesterol ratio, use of lipid modifying medication, and smoking status

Analysis of the association with WMH volume was additionally adjusted for intracranial volume both in model 1 and model 2

**Table 3:** Associations of individual organ bed microvascular dysfunction scores and macrostructural MRI markers of cSVD

	WMH volume		Lacunes		Microbleeds	
	St *β*(95%CI)	*p* value	OR (95%CI)	*p* value	OR (95%CI)	*p* value
Retina	0.021 (− 0.017 to 0.051)	0.32	1.183 (0.918–1.525)	0.19	1.051 (0.895–1.234)	0.54
Kidney	0.041 (− 0.002 to 0.066)	0.06	1.012 (0.819–1.251)	0.91	0.990 (0.848–1.156)	0.90
Skin	0.042 (− 0.009 to 0.073)	0.13	0.858 (0.640–1.149)	0.30	1.098 (0.880–1.369)	0.41
Plasma biomarkers	0.029 (− 0.011 to 0.056)	0.18	0.975 (0.785–1.210)	0.82	0.989 (0.845–1.158)	0.89

Associations of individual organ microvascular function score and structural brain abnormalities in the study population. Point estimates (standardized *β*) and 95% CIs indicate the mean difference in WMH volumes (in log10-transformed ml) per SD increase in the microvascular single organ bed score. Odds ratios with 95% CI represent the risk of the presence of lacunes or cerebral microbleeds. Analyses were adjusted for sex, age, time between baseline and MRI measurement, BMI, diabetes status, office systolic blood pressure, use of antihypertensives, total/HDL cholesterol ratio, use of lipid modifying medication, and smoking status, and intracranial volume in case of WMH volume; model 2

*WMH*, white matter hyperintensity volume; *CI*, confidence interval; *OR*, odds ratio, *SD*, standard deviation

### Extracerebral microvascular function and lacunes and microbleeds

The eMVD compound score was not significantly associated with the presence of lacunes nor microbleeds after adjustment for sex, age, and cardiovascular risk factors (Table 2). None of the individual organ bed eMVD scores was statistically significantly associated with the presence of lacunes or microbleeds (Table 3).

### Additional analyses

Associations remained similar when we replaced BMI with waist circumference, office with ambulatory systolic blood pressure, and diabetes status with fasting plasma glucose or HbA1c levels plus use of glucose-lowering medication (Supplementary Table S4).

Additional correction for alcohol use, diet, and physical activity was performed in a smaller sample because data on diet and physical activity were only available in *n* = 1580. We applied both model 2 and model 2 + additional correction for alcohol use, diet, and physical activity to this sample of 1580 participants, and the regression coefficient *β* was very similar (Supplementary Table S4), implying that alcohol use, diet, and physical activity are not important confounders.

After replacing flicker light-induced retinal arteriolar and venular dilation response with CRAE and CRVE as the retinal component in the compound score, we did not find a significant association with WMH volume, nor lacunes or microbleeds (Supplementary Table S5).

The microvascular dysfunction compound score was not significantly associated with any deep nor any lobar cerebral microbleeds (Supplementary Table S6).

## Discussion

The present study demonstrates that a compound score of eMVD measures is associated with cerebral WMH volume, independent of major cardiovascular risk factors. We found no associations between the compound score and lacunes or microbleeds.

Although measuring cerebral microvascular function in the brain itself is preferable, this is challenging since the limited accessibility of the brain and the size of the affected vessels. Various physiological neuro-imaging techniques have been developed to measure specific functions of the cerebral microcirculation [22] such as blood–brain barrier permeability, microvascular perfusion, and cerebrovascular reactivity [6]. However, these techniques are expensive, complex, only available in specialized neuro-imaging centers, and require substantial image post-processing to obtain quantitative physiological biomarkers, which makes them unsuitable for large scale-studies. As such, eMVD measures could be used as biomarkers for cerebral microvascular health. Our results support the hypothesis that cSVD is part of a systemic disorder of microvascular dysfunction, and a compound score of extracerebral microvascular dysfunction could serve as a proxy for brain MVD.

The eMVD compound score provides a more precise representation of systemic microvascular dysfunction than single organ measures. Previous studies have shown an association between different individual extracerebral microvascular function measures and WMH volume. Albuminuria and lower GFR have both been associated with WMH extensiveness [9, 23, 24]. Previous studies have shown significant associations between different endothelial dysfunction biomarkers and WMH [7, 25–28]. To the best of our knowledge, associations of heat-induced skin hyperemia and cSVD features have not been investigated before. Retinal vascular geometry, microvascular density, and retinal vessel diameters have been shown to be associated with WMH before [29–32]. There are no comparable population-based cohort studies investigating the association of retinal flicker light-induced dilation response, which we consider a more direct functional measure than vessel diameter, and cSVD features. Although retinal vessels are both anatomically and functionally similar to cerebral vessels, and flicker light response can be referred to as a measure of neurovascular coupling, we did not find an association between the retinal vessel flicker light response and cSVD markers.

The fact that we found an association between the compound score and WMH volume, but not with single organ measures, could in part be explained by the fact that the impact of the biologic variability of each individual measure of the compound score is reduced when assuming that there is an overlap between the processes underlying the associations between eMVD measures and the cSVD features [11]. Furthermore, our study population was relatively healthy. The proportion of participants with substantial WMH volumes was small (only 0.4% had WMH volumes ≥ 3% ICV), which might have limited our statistical power to show an association. Moreover, diversity in measuring eMVD measures could have contributed to discrepancies with previous studies. Lastly, many previous studies only adjusted for a limited number of cardiovascular risk factors, whereas we conducted extensive adjustment for relevant cardiovascular risk factors. Our findings support the hypothesis that an overall measure of multiple vascular beds reflects systemic microvascular function better than single organ measures.

Although we demonstrated an association between the eMVD compound score and WMH volume, its magnitude is small, meaning a considerable amount of the variance in WMH is explained by other factors than the eMVD compound score. Unquestionably, peripheral MVD is different from cerebral MVD due to the unique functions of the microcirculation in the brain, such as the blood–brain barrier and neurovascular coupling [33]. Furthermore, there are other factors, including macrovascular changes, contributing to WMH [34].

No associations were found between the microvascular function compound score, nor the single organ microvascular function scores, and the presence of lacunes or cerebral microbleeds. The relatively small number of participants with lacunes (*n* = 96) and cerebral microbleeds (*n* = 219) in our relatively healthy study population would have imposed limitations in multivariate analysis on an association between the microvascular function measures and the presence of lacunes and microbleeds. Possibly, WMH are an earlier occurring marker of cSVD than lacunes or microbleeds. Of note, most previous studies demonstrated an association of extracerebral microvascular dysfunction with deep cerebral microbleeds, but not with lobar cerebral microbleeds, but this could not be shown in our study either.

There are several limitations to this study. One is the cross-sectional design. Longitudinal studies examining changes in microvascular function and progression of cSVD imaging markers would be valuable to infer on the temporality of effects. Second, the study population was relatively healthy and only had subtle cSVD features, imposing limitations in our statistical power to show associations. Third, in our compound score, we used both direct (retinal arteriolar and venular dilation response and heat-induced skin hyperemia) and indirect (abuminuria, GFR, and plasma biomarkers) measures, which could reflect different stadia of microvascular dysfunction. That is, direct measures probably reflect a more subtle form of microvascular dysfunction, which may change acutely and may be reversible, whereas indirect measures reflect a more advanced stage of microvascular disease [11, 35]. However, this might rather have led to an underestimation of the association of the eMVD compound score and MRI markers of cSVD. Fourth, the population included in this study had a more favorable cardiovascular risk profile in comparison with the participants that were not included. This could have resulted in an underestimation of the strength of the association of the eMVD compound score and WMH volume. Fifth, the time passed between extracerebral microvascular function measurements and brain MRI imaging might have influenced the associations observed; nonetheless, we adjusted for this in all analyses and this did not significantly change results. Notwithstanding these limitations, the strengths of our study remain the comprehensive evaluation of the association between eMVD in multiple extracerebral vascular beds and cSVD features on MRI, in a large population-based study, and we extensively adjusted for confounders.

In conclusion, we showed that an eMVD compound score is associated with WMH volume, even in a relatively healthy sample with participants mostly at an early stage of cSVD. Peripheral systemic microvascular dysfunction biomarkers could be useful in the evaluation of the origin of brain microvascular damage. Nonetheless, there remains a need for measuring microvascular function directly in the brain, as a deeper understanding of the pathophysiology of cerebral small vessel disease is of great importance.

## Supplementary Information

Below is the link to the electronic supplementary material.

Supplementary file1 (DOCX 46 KB)

## Data Availability

On request. The online version contains supplementary material at 10.1007/s11357-021-00493-0.
