# Supplementary material for: Extracerebral microvascular dysfunction is related to brain MRI markers of cerebral small vessel disease: The Maastricht Study
Source: GeroScience. 2021 Nov 23;44(1):147–57. doi: 10.1007/s11357-021-00493-0 (PMC8811003; doi:10.1007/s11357-021-00493-0)
Supplement: Supplementary file 1 — Supplementary file1 (DOCX 46 KB) [file 11357_2021_493_MOESM1_ESM.docx]

**Supplementary data

Title:** Extracerebral microvascular dysfunction is related to brain MRI markers of cerebral small vessel disease: The Maastricht Study **Journal:** Geroscience

**Authors:** Maud van Dinther^1,2^, Miranda T. Schram^3,4^, Jacobus F.A. Jansen^4,5^, Walter H. Backes^2,4,5^, Alfons J.H.M. Houben^2,3^, Tos T.J.M. Berendschot ^4,6,7^, Casper G. Schalkwijk^2,3^, Coen D.A. Stehouwer^2,3^, Robert J. van Oostenbrugge^1,2,4^ **^,^** Julie Staals^1,2^

^1^Department of Neurology, Maastricht University Medical Center, Maastricht, The Netherlands
^2^CARIM - School for Cardiovascular Diseases, Maastricht University, Maastricht, The Netherlands
^3^Department of Internal Medicine, Maastricht University Medical Center, Maastricht, The Netherlands
^4^MHeNs - School for Mental Health and Neuroscience, Maastricht University, Maastricht, The Netherlands
^5^Department of Radiology and Nuclear Medicine, Maastricht University Medical Center, Maastricht, The Netherlands
^6^Department of Ophthalmology, Maastricht University Medical Center, Maastricht, The Netherlands
^7^NUTRIM – School of Nutrition and Translational Research in Metabolism, Maastricht University, Maastricht, The Netherlands

**Corresponding author:** M. van Dinther, email address: [maud.van.dinther@mumc.nl](mailto:maud.van.dinther@mumc.nl)

**Item S1. Detailed information on brain MRI sequences**Brain magnetic resonance imaging (MRI) was performed on a 3T MRI scanner (Siemens Magnetom Prisma-fit Syngo MR D13D, Erlangen, Germany) by use of a 64-element head/neck coil for parallel imaging. The MRI protocol consisted of a 3D T1-weighted magnetization prepared rapid acquisition gradient echo (MPRAGE) sequence (TR/TI/TE 2300/900/2.98 ms, 176 slices, 256×240 matrix size, 1.00 mm cubic voxel size); a fluid attenuated inversion recovery (FLAIR) sequence (TR/TI/TE 5000/1800/394 ms, 176 slices, 512×512 matrix size, 0.49×0.49×1.00 mm voxel size); a combined proton density (PD) and T2-weighted turbo spin echo (TSE) pulse sequence (TR/TE1/TE2 3200/9.4/94 ms, 30 slices, 640×540 matrix size, 0.36×0.36×4.00 mm voxel size); and a susceptibility-weighted imaging (SWI) sequence (TR/TE 28/20 ms, 144 slices, 384×312 matrix size, 0.57×0.57×1.00 mm voxel size).

**Item S2. Methodology of acquiring retinal vessel diameters**We measured retinal vessel diameters by means of fundus photography using a focus, shot, and tracker fundus camera (model AFC-230; Nidek Co. Ltd., Aichi, Japan), as previously described.(1) Briefly, fundus photographs of both eyes are made in 45 degrees of at least 3 fields: 1 field centered on the optic disc, 1 field centered on the macula, and 1 field temporal field positioned 1 disc diameter from the center of the macula. Static retinal vessel analysis is performed with the RHINO software developed by the RetinaCheck Project group at the Eindhoven University of Technology (Eindhoven, the Netherlands). Retinal vessel diameters were measured 0.5-1.0 disc diameter away from the optic disc margin and 1.0-1.5 disc diameter away from the fovea (macula) center (zone A). The scale factor is based on the optic disc diameter, which is assumed to be 1,800 µm.(2) Retinal vessel diameters are presented as central retinal arteriolar equivalent and central retinal venular equivalent. Central retinal arteriolar equivalent and central retinal venular equivalent represent the equivalent single-vessel parent diameters for the 6 largest arterioles and largest venules in the region of interest, respectively. The calculations are based on the improved Knudtson-Hubbard formula.(3)


References

1. Li W, Schram MT, Sorensen BM, van Agtmaal MJM, Berendschot T, Webers CAB, et al. Microvascular Phenotyping in the Maastricht Study: Design and Main Findings, 2010-2018. Am J Epidemiol. 2020;189(9):873-84.

2. Williams TD, Wilkinson JM. Position of the fovea centralis with respect to the optic nerve head. Optom Vis Sci. 1992;69(5):369-77.

3. Knudtson MD, Lee KE, Hubbard LD, Wong TY, Klein R, Klein BE. Revised formulas for summarizing retinal vessel diameters. Curr Eye Res. 2003;27(3):143-9.

**Table S1.** General characteristics of the study population with and without brain MRI and at least 3 extracerebral microvascular function measures available

| Characteristic | Study population (N = 1872) | No brain MRI or <3 eMVD measures available  (N = 1579) | p value |
| --- | --- | --- | --- |
| Age [y] | 59 ± 8 | 60 ± 8 | <0.01 |
| Sex [% women] | 48.8 | 48.4 | 0.70 |
| BMI [kg/m^2^] | 26.5 ± 4.2 | 27.7 ± 4.9 | <0.01 |
| Diabetes[% none/prediabetes/diabetes] | 59.9/15.6/24.5 | 50.8/13.8/35.4 | <0.01 |
| Office systolic blood pressure [mmHg] | 134 ± 17 | 136 ± 19 | <0.01 |
| Blood pressure lowering medication % | 35.5 | 46.1 | <0.01 |
| Smoking, never/former/current % | 37.6/50.9/11.6 | 30.8/52.7/16.5 | <0.01 |
| Total/HDL cholesterol ratio | 3.6 ± 1.2 | 3.7 ± 1.2 | <0.01 |
| Lipid modifying medication % | 31.5 | 42.5 | <0.01 |
| Alcohol use, none/low/high % | 17.1/56.3/26.6 | 20.6/54.5/24.9 | <0.01 |
| Physical activity [hours/week] | 14 ± 8 | 14 ± 9 | 0.96 |
| DHD index | 84 ± 14 | 82 ± 15 | <0.01 |
| History of cardiovascular disease % | 11.8 | 22.8 | <0.01 |
| History of stroke [self reported/stroke on MRI] % | 1.9/1.9 | 3.9/2.6 | <0.01 / 0.05 |

Data are presented as means ± SD, median (interquartile range) or percentages and stratified for availability of data of MRI and at least 3 extracerebral microvascular function measures. Independent t-tests (continuous variables) and X^2^ tests (categorical variables) were used to compare groups.
eMVD indicates extracerebral microvascular dysfunction; BMI, body mass index; HDL, high density lipoprotein; DHD index, Dutch Healthy Diet index; SD, standard deviation.

**Table S2.** Association of the extracerebral microvascular dysfunction compound score and WMH volume. Supplement to Table 2: Full model 1

| **Independent variables in the model** | **WMH volume** | |
| --- | --- | --- |
|  | St β (95%CI) | p-value |
| eMVD compound score | 0.099 (0.046 – 0.113) | < 0.01 |
| Female sex | 0.118 (0.071 – 0.165) | < 0.01 |
| Age | 0.471(0.430 – 0.512) | < 0.01 |
| Time between eMVD measurements and MRI measurements | 0.104 (0.065 – 0.144) | < 0.01 |
| Intracranial volume | 0.159 (0.159 - 0.159) | < 0.01 |

Independent associations of all variables in model 1 with WMH volume (in log10-transformed ml). Point estimates (standardized β) and 95% CIs indicate the mean difference in WMH volume (in log10-transformed ml) per SD increase in the independent variable.
WMH indicates white matter hyperintensity volume; eMVD, extracerebral microvascular dysfunction; CI, confidence interval; SD standard deviation

**Table S3.** Association of the extracerebral microvascular dysfunction compound score and WMH volume.
Supplement to Table 2: Full model 2

| **Independent variables in the model** | **WMH volume** | |
| --- | --- | --- |
|  | St β (95%CI) | p-value |
| eMVD compound score | 0.057 (0.010 – 0.081) | 0.01 |
| Female sex | 0.152 (0.102 – 0.203) | < 0.01 |
| Age | 0.431 (0.380 – 0.472) | < 0.01 |
| Time between eMVD measurements and MRI measurements | 0.096 (0.056 – 0.135) | < 0.01 |
| BMI | -0.042 (-0.089 – 0.000) | 0.07 |
| DM status No DM vs. pre-DM  No DM vs. DM type 2  No DM vs. other types of DM | 0.032 (-0.009 – 0.074)  0.075 (0.026 – 0.124)  0.025 (-0.014 – 0.064) | 0.13  < 0.01  0.21 |
| Office systolic blood pressure | 0.061 (0.020- 0.102) | 0.01 |
| Use of antihypertensives | 0.067 (0.020 – 0.115) | 0.01 |
| Total/HDL cholesterol ratio | -0.026 (-0.069 – 0.017) | 0.25 |
| Use of lipid modifying medication | 0.027 (-0.022 – 0.076) | 0.29 |
| Smoking status  Never vs. former  Never vs. current | 0.028 (-0.014 – 0.083)  0.064 (0.022 – 0.251) | 0.19  <0.01 |
| Intracranial volume | 0.181 (0.181 – 0.181) | < 0.01 |

Independent associations of all variables in model 2 with WMH volume (in log10-transformed ml). Point estimates (standardized β) and 95% CIs indicate the mean difference in WMH volume (in log10-transformed ml) per SD increase in the independent variable.
WMH indicates white matter hyperintensity volume; eMVD, extracerebral microvascular dysfunction; BMI, body mass index; DM, diabetes mellitus; HDL, high density lipoprotein; CI, confidence interval; SD standard deviation

**Table S~~2~~4.** ~~Sensitivity~~Additional analyses of the association of the extracerebral microvascular dysfunction compound score and WMH volume

| **Covariates in the model** | WMH volume | | |
| --- | --- | --- | --- |
|  | St β (95%CI) | p-value | Data available in n= |
| BMI substituted to: waist circumference (model 2) | 0.055 (0.008 - 0.079) | 0.02 | 1846 |
| Office BP substituted to: 24h ambulatory BP (model 2) | 0.052 (0.004 - 0.079) | 0.03 | 1619 |
| DM status substituted to: fasting plasma glucose and glucose lowering medication (model 2) | 0.054 (0.008 - 0.079) | 0.02 | 1846 |
| DM status substituted to: HbA1c and glucose lowering medication (model 2) | 0.059 (0.012 - 0.082) | 0.01 | 1843 |
| Model 2 + additional adjustment for alcohol use, physical activity and diet | 0.039 (-0.008 – 0.071) | 0.12 | 1580 |
| Model 2 limited to n=1580 subjects with available data on alcohol use, physical activity and diet | 0.038 (-0.008 – 0.070) | 0.13 | 1580 |

~~Sensitivity~~Additional analyses of the associations of the microvascular dysfunction compound score and WMH volume adjusted for sex, age, time between baseline and MRI measurement, BMI, diabetes status, office systolic blood pressure, use of antihypertensives, total/HDL cholesterol ratio, use of lipid modifying medication, smoking status, and intracranial volume. Sensitivity analyses included subsequently substituting BMI for waist circumference, office blood pressure for 24-h ambulatory blood pressure, DM status for HbA1c + glucose lowering medication, and DM status for fasting plasma glucose + glucose lowering medication, and additional adjustment for alcohol use, physical activity and diet.
Point estimates (standardized β) and 95% CIs indicate the mean difference in WMH volume (in log10-transformed ml) per SD increase in the microvascular compound score.
WMH indicates white matter hyperintensensity volume; BMI, body mass index; BP, blood pressure; DM, diabetes mellitus; HbA1c, Hemoglobin A1c; CI, confidence interval; SD standard deviation

**Table S~~3~~5.** Analyses of the association of the extracerebral microvascular dysfunction compound score, in which flicker light-induced arterioral and venular dilation response were replaced for CRAE and CRVE, and cSVD features

|  | **WMH volume** | | **Lacunes** | | **Microbleeds** | |
| --- | --- | --- | --- | --- | --- | --- |
|  | St β (95%CI) | p-value | OR (95%CI) | p-value | OR (95%CI) | p-value |
| **eMVD compound score**  **Model 2** | 0.040 (-0.003 – 0.067) | 0.07 | 0.989 (0.784 – 1.247) | 0.99 | 0.991 (0.841 – 1.167) | 0.91 |

Associations of the microvascular dysfunction compound score, in which flicker light-induced retinal and venular dilation response were replaced with CRAE and CRVE, and structural brain abnormalities in the study population. Analyses were adjusted for sex, age, time between the extracerebral microvascular function measures and MRI measurement, BMI, diabetes status, office systolic blood pressure, use of antihypertensives, total/HDL cholesterol ratio, use of lipid modifying medication, and smoking status (model 2). For analysis of the association with WMH volume we additionally adjusted for intracranial volume. Point estimates (standardized β) and 95% CIs indicate the mean difference in WMH volume (in log10-transformed ml) per SD higher microvascular compound score. Odds ratios with 95% CI represent the risk of the presence of lacunes or cerebral microbleeds.
WMH indicates white matter hyperintensity volume; CI, confidence interval; OR, odds ratio, eMVD extracerebral microvascular dysfunction, SD standard deviation

**Table S~~4~~6.** Analyses of the association of the extracerebral microvascular dysfunction compound score and microbleeds

|  | **Deep microbleeds** | | **Lobar microbleeds** | |  |
| --- | --- | --- | --- | --- | --- |
|  | OR (95%CI) | p-value | OR (95%CI) | p-value | |
| **eMVD compound score**  Model 1  Model 2 | 0.885 (0.568 – 1.379)  0.879 (0.542 – 1.424) | 0.59  0.60 | 1.052 (0.883 – 1.253)  1.013 (0.838 – 1.225) | 0.57  0.89 | |

Associations of the microvascular dysfunction compound score and microbleeds in the study population. Odds ratios with 95% CI represent the risk of the presence of deep and lobar cerebral microbleeds.
OR indicates odds ratio; eMVD extracerebral microvascular dysfunction.
Model 1: adjustment for sex, age and time between the extracerebral microvascular function measures and MRI measurement .
Model 2: model 1 additionally adjusted for BMI, diabetes status, office systolic blood pressure, use of antihypertensives, total/HDL cholesterol ratio, use of lipid modifying medication, and smoking status.

**Figure S1**Derivation of the final study populations
* missing data on brain on MRI including excluded MRI scans due to pathology (n=2), metal artifacts (n=1) and insufficient scan quality (n=8)
